# Disease of the Parotid Gland in Typhoid Fever

**Published:** 1873-02-01

**Authors:** 


DISEASE OF THE PAROTID GLAND IN TYPHOID FEVER.—Inflammation, suppuration, and gangrene, affecting the parotid region, in the progress of low forms of fever, is not as rare as might be inferred from reading the interesting cases reported by Dr. Kelsey in the present number. We have met with several cases of the kind. While the shreds and masses of sloughing tissue should be removed as fast as they become actually loose, it is not necessary to hasten the process violently.

